# Influence of nutritional status and body composition on postoperative events and outcome in patients treated for primary localized retroperitoneal sarcoma

**DOI:** 10.2478/raon-2024-0013

**Published:** 2024-02-21

**Authors:** Manuel Ramanovic, Marko Novak, Andraz Perhavec, Taja Jordan, Karteek Popuri, Nada Rotovnik Kozjek

**Affiliations:** Biotechnical Faculty, University of Ljubljana, Ljubljana, Slovenia; Department of Surgical Oncology, Institute of Oncology Ljubljana, Ljubljana, Slovenia; Medical Faculty, University of Ljubljana, Ljubljana, Slovenia; Department for Radiology, University Medical Centre Ljubljana, Ljubljana, Slovenia; Department of Computer Science, Memorial University of Newfoundland, Newfundland, Canada; Department of Clinical Nutrition, Institute of Oncology Ljubljana, Ljubljana, Slovenia

**Keywords:** body composition, myopenia, cancer cachexia, myosteatosis, obesity, retroperitoneal sarcoma

## Abstract

**Background:**

Retroperitoneal sarcomas (RPS) are rare tumours of mesenchymal origin, commonly presented as a large tumour mass at time of diagnosis. We investigated the impact of body composition on outcome in patients operated on for primary localized RPS.

**Patients and methods:**

We retrospectively analysed data for all patients operated on for primary RPS at our institution between 1999 and 2020. Preoperative skeletal muscle area (SMA), visceral and subcutaneous adipose tissue area (VAT and SAT) and muscle radiation attenuation (MRA) were calculated using computed tomography scans at the level of third lumbar vertebra. European Working Group on Sarcopenia in Older People (EWGSOP2) criteria were applied to define myopenia. Using maximum log-rank statistic method we determined the optimal cut-off values of body composition parameters. Myosteatosis was defined based on determined MRA cut-offs.

**Results:**

In total 58 patient were eligible for the study. With a median follow-up of 116 months, the estimated 5-year overall survival (OS) and local-recurrence free survival (LRFS) were 66.8% and 77.6%, respectively. Patients with myopenia had significantly lower 5-year OS compared to non-myopenic (p = 0.009). Skeletal muscle index and subcutaneous adipose tissue index predicted LRFS on univariate analysis (p = 0.052 and p = 0.039, respectively). In multivariate analysis high visceral-to-subcutaneous adipose tissue area ratio (VSR) independently predicted higher postoperative complication rate (89.2% *vs*. 10.8%, p = 0.008). Myosteatosis was associated with higher postoperative morbidity.

**Conclusions:**

Myopenia affected survival, but not postoperative outcome in RPS. Visceral obesity, VSR (> 0.26) and myosteatosis were associated with higher postoperative morbidity. VSR was better prognostic factor than VAT in RPS.

## Introduction

Retroperitoneal sarcomas (RPS) are soft tissue tumours of mesenchymal origin accounting for approximately 15% of all sarcomas and less than 1% of all tumour malignancy.^1,2,3^ Most patients develop large tumour mass before diagnosis is clinically confirmed. Imaging techniques, CT and MRI are primarily used in clinical evaluation of RPS.^1,4^ Surgical resection with removal of all gross disease is the cornerstone of curative therapy and optimal results are achieved with *en bloc* resection at the time of primary presentation.^4,5^ The role of radiation therapy and chemotherapy in management of RPS is still under investigation. Following the STRASS trial and STREXIT study, it seems that preoperative radiotherapy might influence the local control in liposarcoma (LPS) patients, while the evaluation of chemotherapy remains ongoing for high-grade LPS and leiomyosarcoma.^6,7,8^ There are no currently available data supporting the use of routine neoadjuvant or adjuvant chemotherapy for these patients.^1^ Optimal management is achieved in specialized sarcoma centres^4,9^ with multidisciplinary approach.^10,11,12,13^ Institute of Oncology Ljubljana is the only referral sarcoma centre in Slovenia.^14^

Body composition changes are related to nutrition status and associated with perioperative outcome and influence management of surgical oncology patients.^15^ Ongoing catabolic processes, systemic inflammation, as well as decreased protein synthesis, as part of often presented cancer-associated cachexia, together contribute to loss of lean body mass and pose a risk of malnutrition in sarcoma patients.^16,17,18^ Sarcopenia is a clinical syndrome in which involuntary loss of skeletal muscle mass and function is progressive and generalized, together or without increased fat mass.^19,20^ Another clinically important body composition abnormality, myosteatosis, is characterized by excess accumulation of adipose tissue within muscle, resulting in impaired muscle strength and physical ability, as well as increased frailty.^21,22^ Recent studies demonstrated that both sarcopenia and myosteatosis pose a greater risk for postoperative complications and decrease overall survival (OS) in a variety of different cancers, including soft tissue sarcomas.^22,23,24,25,26,27^ Visceral obesity (VO) is the fat accumulation in visceral adipose tissue and serves as a clinical marker for adiposopathy.^28^ Number of recent studies reported that VO is more reliable clinical marker for predicting outcome than traditional view on obesity defined by BMI.^29,30,31,32,33^ The useful predictor of VO is visceral-to-subcutaneous adipose tissue area ratio (VSR), and high VSR is associated with poor oncologic outcome.^34,35,36,37^ Also, another body composition abnormality, the new concept of sarcopenic obesity (SO), a combination of excess adiposity and sarcopenia, seems to have powerful negative prognostic impact in oncology treatment and is gaining increased attention in cancer research.^38,39^ Loss of muscle mass or myopenia is a critical determinant of sarcopenia.

CT has been shown to be a precise and feasible method to evaluate body composition parameters.^40,41,42,43^ There is a lack of literature data regarding the impact of body composition on postoperative and oncologic outcome in patients operated on for primary RPS.

The aim of our study is to investigate the impact of low muscle mass or myopenia, myosteatosis, visceral obesity and cancer cachexia on OS, local recurrence-free survival (LRFS) and postoperative morbidity in patients operated for primary localized RPS. Additionally, we aimed to investigate the predictive value of preoperative body composition parameters for OS, LRFS and postoperative morbidity.

## Patients and methods

### Study design and population

**FIGURE 1.**
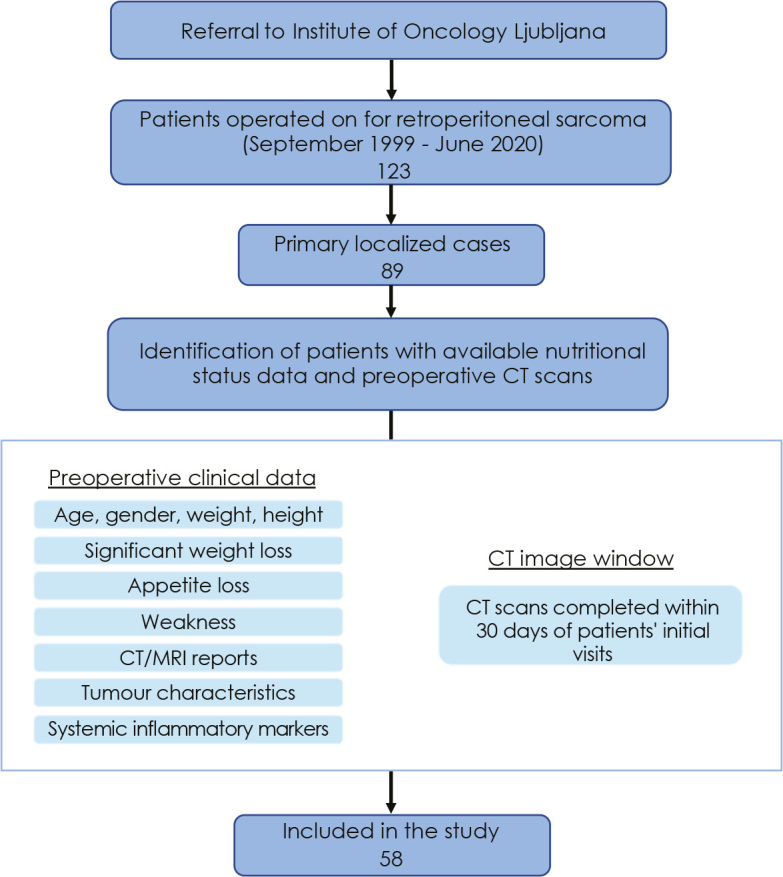
Patients' flow diagram. Out of 123 patients, 58 (47.1%) with primary localized retrperitoneal sarcomas (RPS) were included in the study and 65 were excluded. 34 (27.6%) were excluded as they presented as primary metastatic cases (6), locally recurrent cases (14) or cases with residual disease after operation elsewhere (14), and 31 patients (25.2%) were excluded as they had CT performed > 30 days from initial assessment or CT image was not technically adequate for analysis

Retrospective study was conducted on patients operated on for primary RPS at Department of Surgical Oncology at the Institute of Oncology Ljubljana between September 1999 and June 2020 (Figure 1). A total of 58 patients met the inclusion criteria, 24 females (41.4%) and 34 males (58.6%). The Slovenian National Medical Ethical Committee (decision number: 0120-530/2020/3), Institutional Review Board (ERID-KSOPKR-0081/2020) and Institutional Ethical Committee (ERIDEK-0079/2020) approved the study. Due to the retrospective nature of the study the need to obtain informed consent from participants was waived.

### Clinical data collection

Patient's histories including anesthesiologic pre-operative reports, operative reports, hospital records, and follow-up data were reviewed. Clinical and pathological data were collected (Figure 1). Postoperative complications were evaluated in accordance with Clavien–Dindo classification.^44^ Tumour features of interest were as follows: histopathological diagnosis, stage (according to American Joint Committee on Cancer [AJCC] 8th Edition), grade (according to National Federation of Centers for the Fight Against Cancer grading system)^45^ and tumour size (largest diameter value). Resection quality was recorded as either complete (R0), incomplete (R1) with microscopic involvement of resection margins or macroscopic residual tumour (R2).

### Body composition assessment

The assessment of body composition was conducted using images from CT scans taken within 30 days preoperatively at the level of the third lumbar vertebra using the “Automated Body Composition Analyzer using Computed tomography image Segmentation” (ABACS) software.^46^ This method uses predefined CT Hounsfield units (HU) values to recognize different tissues. The CT HU thresholds were 29 to 150 for skeletal muscles, 190 to 30 for subcutaneous adipose tissue, and 150 to 50 for visceral adipose tissue. The following body composition parameters were measured: total cross-sectional skeletal muscle area (SMA, cm^2^), subcutaneous adipose tissue area (SAT, cm^2^) and visceral adipose tissue area (VAT, cm^2^). After normalization by patient's height (m^2^), we used these parameters as lumbar skeletal muscle index (SMI, cm^2^/m^2^), subcutaneous adipose tissue index (SATI, cm^2^/m^2^), and visceral adipose tissue index (VATI, cm^2^/m^2^). VSR was calculated by dividing VAT by SAT. To assess the muscle density and myosteatosis, skeletal muscle radiation attenuation (MRA) has also been recorded in HUs. All measurements were performed by experienced researcher, accredited for complex image analysis and segmentation techniques. Additionally, we used previously reported and validated equations to calculate appendicular skeletal muscle index (ASMI), lean body mass (LBM) and fat mass (FM)^26,29,43,47^:

**ASMI** (kg/m^2^) = 0.11 × SMI (cm^2^/m^2^) + 1.17
**LBM** (kg) = 0.030 × Lean Tissue Area (cm^2^) + 6.06
**FM** (kg) = 0.042 × Total Fat Area (cm^2^) + 11.2

Based on a single abdominal CT image per patient, LBM and FM properly reflect appropriate dual-energy X-ray absorptiometry (DXA) derived whole-body fat-free mass (FFM) and whole-body fat mass (FM), respectively.

### Assessment of myopenia, myopenic and visceral obesity, myosteatosis and cancer cachexia

Myopenia was defined based on the new recommendations of The European Working Group on Sarcopenia in Older People (EWGSOP2), as follows: SMI < 43 cm^2^/m^2^ for men with BMI < 25, SMI < 53 cm^2^/m^2^ for men with BMI ≥ 25, and SMI < 41 cm^2^/m^2^ for women.^48^

Muscle mass in patients with obesity was assessed according to The European Society for Clinical Nutrition and Metabolism (ESPEN) and the European Association for the Study of Obesity (EASO) consensus report for sarcopenic obesity.^39^ Previously reported diagnostic criteria for visceral obesity were applied: VAT > 163.8 cm^2^ for men and VAT > 80.1 cm^2^ for women.^29,39,47^

Preoperative cancer cachexia was defined using Fearon *et al*. criteria.^49^

In order to establish optimal cut-off values for SMI, VATI, SATI, VSR and MRA which would best reflect our study cohort in relationship to defined outcome (maximum OS), we performed optimal stratification analysis based on maximally selected rank statistics using *maxstat* package implemented in R statistics.^50,51^ This approach is widely used and validated in cancer patients.^26,52,53,54,55^ The presence of myosteatosis was then confirmed based on established optimal threshold for MRA: < 35.88 HU in patients with a BMI ≥ 25 kg/m^2^ and < 47.41 HU in those with a BMI < 25 kg/m^2^.

### Survival and statistical analysis

Final survival follow-up time was set as last follow-up date in the study period or the event of death. OS was defined as time between the date of the operation and date of death from any cause or last follow-up. LRFS was defined as the time interval between operation date and date of first documented local progression, and instances involving deaths without evidence of disease and the occurrence of distant metastases considered as competing events. Survival curves were estimated using Kaplan-Meier method. Log-rank test, linear regression and Cox proportional hazard regression models were used to analyse the relationship between clinicopathological parameters and survival. Hosmer-Lemeshow test assessed the prediction accuracy (goodness of fit) of regression models. Hazard ratios (HRs) and 95% confidence intervals (CIs) were obtained.

In addition to body composition parameters, following known prognostic factors or other clinical features were considered: age, gender, American Society of Anesthesiologists (ASA) classification, Albumin level (g/dL), C-reactive protein (mg/L), neutrophil-to-lymphocyte ratio (NLR), preoperative radiotherapy, tumour size (cm), and intraoperative blood loss (ml). Results were statistically significant if two-sided p value < 0.05 was achieved. R statistical software (version 4.2.1, R core Team) was used.

## Results

### Demographic and clinical characteristics

Out of 89 primary localized RPS cases, clinical and pathological characteristics and preoperative abdominal CT scans technically adequate for analysis were available for 58 patients, representing the final study cohort (Figure 1). The demographic and clinical characteristics of the patients are provided in Table 1. In the cohort, 34 (58.6%) were males and 24 (41.4%) were females. Median age at diagnosis was 61.0 (46.0–67.0). Loss of muscle mass according to EWGSOP2 criteria, was present in 19 patients (32.8%). Applying our cut-off values for low SMI, comparable number of myopenic patients was detected, 12 males (66.7%) and 6 females (33.33%). Significant difference between myopenic and non-myopenic group was detected in tumour size (median 26 *vs*. 17.5 cm, p = 0.005), SMA (median 115.8 *vs*. 149.9 cm^2^, p = 0.019), SMI (median 41 *vs*. 53.5 cm^2^/m^2^, p < 0.001), SATI (46.8 *vs*. 64.6 cm^2^/m^2^, p = 0.048) and VATI (20.6 *vs*. 42.3 cm^2^/m^2^, p = 0.025) (Table 2). There was no significant difference in clinical management among myopenic and nonmyopenic group.

**TABLE 1.** Clinical characteristics of study population

**Clinical characteristic (N = 58)**	**Median (IQR); n (%)**
Age, years	61.0 (46.0 – 67.0)
Gender	
Male	34 (58.6%)
Female	24 (41.4%)
ASA grade	
1	9 (16%)
2	30 (52%)
3	16 (28%)
4	3 (5.2%)
Baseline albumin, g/L	41.0 (34.2 – 45.0)
Baseline C-reactive protein, mg/L	13.5 (2.0 – 66.5)
Neutrophil-lymphocyte ratio	3.3 (2.1 – 4.7)
Body Mass Index, kg/m^2^	26.0 (24.7 – 29.7)
**Nutrition and body composition characteristics**	
Nutritional team support before operation	28(48.3%)
Skeletal Muscle Area, cm^2^	45.5 (115.9 – 170.1)
Visceral Fat Area, cm^2^	104.5 (53.6 – 168.7)
Subcutaneous Adipose Tissue Area, cm^2^	167.9 (127.9 – 231.6)
Total Fat Area, cm^2^	23.6 (19.8 – 29.1)
Total Body Fat, %	30.6 (26.8 – 32.4)
Lean Body Mass, kg	52.7 (50.0 – 57.2)
Skeletal Muscle Index, cm^2^/m^2^	50.2 (44.0 – 55.6)
Appendicular Skeletal Muscle Index, cm^2^/m^2^	6.70 (6.00 – 7.3)
Myopenia based on estimated cut-off value for SMI^a^	18 (31.0%)
Myopenia based on EWGSOP2 criteria for SMI	19 (32.8%)
Cancer cachexia	13 (22.4%)
Visceral obesity	21 (36.2%)
Myopenic obesity	4 (6.9%)
Myosteatosis^a^	37 (63.7%)
**Pathologic characteristics and postoperative outcome data**	
Postoperative (90 day) complication rate	37 (64%)
Abdominal complication	24 (41%)
Systemic complication	17 (29%)
Abdominal and systemic complications	5 (9.0%)
Clavien-Dindo > IIIa	
Yes	17 (29%)
No	41 (71%)
Comprehensive Complication Index	20.92 (0.0–32.55)
Histologic type	
Liposarcoma	35 (60%)
Leiomyosarcoma	9 (16%)
Pleomorphic sarcoma	1 (1.7%)
Other	13 (22%)
Tumour size, cm	20 (11–30)
FNCLCC grade	
1	15 (26%)
2	11 (19%)
3	23 (40%)
Unknown	9 (16%)
Stage AJCC (8^th^ edition)	
1A	1 (1.7%)
1B	23 (40%)
3A	6 (10%)
3B	28 (48%)
Completeness of surgical resection	
R0	47 (81%)
R1/R2	11 (19%)

AJCC = The American Joint Committee on Cancer; ASA = American Society of Anesthesiologists classification; EWGSOP2 = The European Working Group on Sarcopenia in Older People; FNCLCC = Fédération Nationale des Centres de Lutte Contre Le Cancer

Summary for continuous variables is presented as median (interquartile range) and the statistical test is Kruskal-Wallis/Mann-Whitney;

a cut-off values displayed in Table 3

**TABLE 2.** Comparison of clinical and body composition parameters between myopenic and non-myopenic patients (EGSWOP2 criteria)

**Clinicopathological factor**	**Level^a^**	**Myopenic^b^**	**Non Myopenic^b^**	**p**
Age, years	Median (IQR)	66.0 (50.5–71.5)	61.0 (46.0–64.8)	0.236
Gender	Male	11(57.9)	23 (60.5)	1
	Female	8(42.1)	15 (39.5)	
ASA Grade, 2–3 *vs*. 1	1	3 (15.8)	6 (15.8)	1
	2–3	16 (84.2)	32 84.2)	
FNCLCC Grade	1	5 (29.4)	10 (32.3)	0.547
	2	5 (29.4)	5 (16.1)	
	3	7 (41.2)	16 (51.6)	
	(Missing)	7 (18.4)	2 (10.5)	
Tumour size, cm	Median (IQR)	26.0 (20.5–34.0)	17.5 (10.0–24.8)	0.005
Clavien-Dindo > IIIa	Yes	3 (15.8)	14 (36.8)	0.183
	No	16 (84.2)	24 (63.2)	
Neutrophil-lymphocyte ratio	Median (IQR)	3.9 (2.4–4.7)	3.0 (2.1–4.7)	0.504
Baseline albumin, g/L	Median (IQR)	40.0 (32.0–42.5)	43.0 (35.0–45.8)	0.232
Baseline C-reactive protein, mg/L	Median (IQR)	44.0 (7.5–99.5)	6.0 (2.0–45.0)	0.088
Haemoglobin level, g/L	Median (IQR)	128.0 (101.5–136.5)	132.5 (115.2–145.8)	0.141
Preoperative radiotherapy	No	19 (100.0)	37 (97.4)	1
	Yes	0 (0.0)	1 (2.6)	
Resection status	R0	17 (89.5)	29 (76.3)	0.406
	R1	2 (10.5)	9 (23.7)	
Intraoperative blood loss, ml	Median (IQR)	1300.0 (425.0–4100.0)	1350.0 (500.0–2075.0)	0.78
Stage AJCC, 8th edition	1A–1B	7 (36.8)	17 (44.7)	0.776
	3A–3B	12 (63.2)	21 (55.3)	
Histology subtype	Pleomorphic	1 (5.3)	0 (0.0)	0.184
	Liposarcoma	14 (73.7)	21 (55.3)	
	Leiomyosarcoma	2 (10.5)	6 (15.8)	
	Other	2 (10.5)	11 (28.9)	
Nutrition team before surgery	Yes	12 (63.2)	16 (42.1)	0.223
	No	7 (36.8)	22 (57.9)	
Length of hospital stay, days	Median (IQR)	20.0 (11.0–28.8)	15.0 (11.5–27.0)	0.593
Visceral obesity	Yes	4 (21.1)	16 (42.1)	0.202
	No	15 (78.9)	22 (57.9)	
Myosteatosis	Yes	16 (84.2)	21 (56.8)	0.079
	No	3 (15.8)	16 (43.2)	
Cancer cachexia	No	12 (63.2)	31 (83.8)	0.163
	Yes	7 (36.8)	6 (16.2)	
Body Mass Index, kg/m^2^	Median (IQR)	25.7 (23.3–27.2)	26.9 (24.8–30.9)	0.071
Skeletal Muscle Area, HU	Median (IQR)	115.8 (106.5–153.3)	149.9 (130.6–177.1)	0.019
Skeletal Muscle Index, cm^2^/m^2^	Median (IQR)	41.0 (38.3–46.8)	53.5 (46.2–58.8)	< 0.001
Muscle Radiation Attenuation, HU	Median (IQR)	35.6 (31.8–43.2)	38.1 (29.9–42.2)	0.959
Subcutaneous Adipose Tissue Area, cm^2^	Median (IQR)	156.4 (103.2–194.4)	185.4 (131.4–254.1)	0.078
Visceral Adipose Tissue Area, cm^2^	Median (IQR)	64.6 (38.1–131.6)	125.5 (66.1–201.7)	0.07
Visceral-to-subcutaneous adipose tissue area ratio	Median (IQR)	0.5 (0.2–0.9)	0.8 (0.3–1.1)	0.393
Body fat, %	Median (IQR)	28.3 (21.3–31.6)	31.1 (28.1–33.3)	0.024
Lean Body Mass, kg	Median (IQR)	52.5 (50.4–57.7)	52.8 (49.8–56.4)	0.684
Subcutaneous Adipose Tissue Index, cm^2^/m^2^	Median (IQR)	46.8 (30.6–67.2)	64.6(43.9–95.0)	0.048
Visceral Adipose Tissue Index, cm^2^/m^2^	Median (IQR)	20.6 (13.2–43.8)	42.3 (24.2–63.6)	0.025

a Summary for continuous variables is median (interquartile range) and the statistical test is Kruskal-Wallis/Mann-Whitney;

b Median (IQR); n (%);

AJCC =The American Joint Committee on Cancer; ASA = American Society of Anesthesiologists; EGSWOP2 = The European Working Group on Sarcopenia in Older People; FNCLCC = Fédération Nationale des Centres de Lutte Contre Le Cancer; HU = Hounsfield units

**TABLE 3.** Results of optimal stratification analysis for body composition parameters

**BMI, kg/m^2^**	**Skeletal Muscle Index^a^, cm^2^/m^2^**		**Visceral Adipose Tissue Index^b^, cm^2^/m^2^**		**Subcutaneous Adipose Tissue Index^b^, cm^2^/m^2^**		**Visceral to subcutaneous ratio^c^**		**Muscle Radiation Attenuation^d^, HU**	

	**Male**	**Female**	**Male**	**Female**	**Male**	**Female**	**Male**	**Female**	**Male**	**Male**
**< 25**	49.21	49.21	61.38	25.55	49.23	86.89	0.26		47.41	
**≥ 25**	49.90	50.64							35.88	

BMI = body mass index; HU = Hounsfield Unit;

a adjusted for gender and BMI;

b adjusted for gender only;

c cut-off determined on the level of whole cohort, not stratified for BMI nor gender;

d adjusted for BMI only.

Males had significantly higher mean values of SMA (163.7 *vs*. 120 cm^2^, p < 0.001), SMI (52.8 *vs*. 45.3 cm^2^/m^2^, p = 0.006), VAT (153.9 *vs*. 96 cm^2^, p = 0.045) and VSR (1.0 *vs*. 0.5, p = 0.001), while in females SAT (227.2 *vs*. 155.1 cm^2^, p = 0.003) and SATI (85.0 *vs*. 49.8 cm^2^/m^2^, p = 0.001) were significantly higher (Supplementaly Table 1).

**FIGURE 2.**
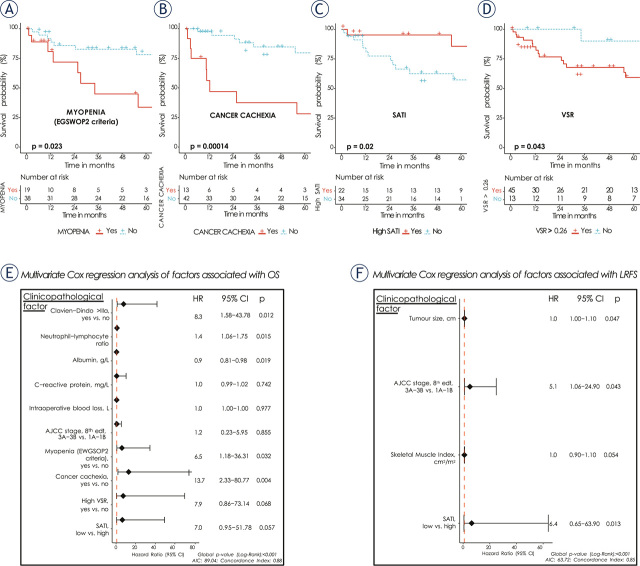
Kaplan-Meier curves for OS **(A–D)** and forest plots of multivariate Cox regression analysis of factors associated with OS **(E)** and LRFS **(F)**. Kaplan-Meier curves for OS according to presence of: **(A)** myopenia based on EWGSOP2 criteria (red = myopenic, blue = non-myopenic), **(B)** cancer cachexia (red = cachectic, blue = non-cachectic), **(C)** high SATI (red = SATI above estimated cohort cut-off, blue = SATI below estimated cohort cut-off) and **(D)** high VSR (red = VSR > 0.26, blue = VSR < 0.26); EWGSOP2 = The European Working Group on Sarcopenia in Older People revised criteria from 2018; SATI = Subcutaneous Adipose Tissue Index, cm^2^/m^2^; SMI = Skeletal Muscle Index; VSR = Visceral-to-subcutaneous adipose tissue area ratio; OS = Overall survival; LRFS = Local recurrence-free survival; HR = Hazard ratio; AIC = Akaike Information Criterion

**FIGURE 3.**
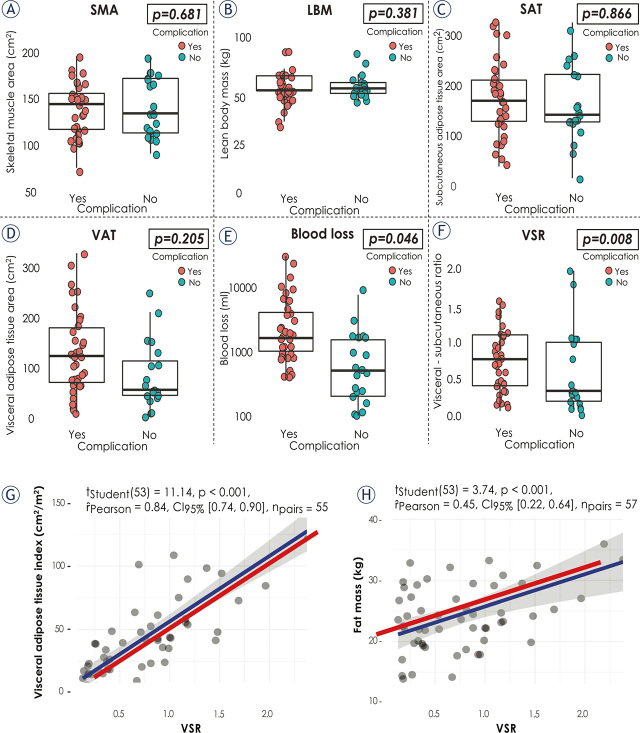
Association between overall morbidity following surgery for primary RPS and body composition **(A–F)** and linear correlation analysis between VSR and VATI **(G)** and VSR and fat mass **(H)**. VSR **(F)** and intraoperative blood loss **(E)** independently predicted worse postoperative outcome. In multivariate analysis skeletal muscle index (SMA), lean body mass (LBM), subcutaneous adipose tissue area (SAT) and visceral adipose tissue area (VAT) were not associated with statistically higher overall postoperative morbidity **(A–D)**. VATI = Visceral Adipose Tissue Index; VSR = Visceral-to-subcutaneous adipose tissue area ratio. ȓ_Pearson_ = Pearson Correlation Coefficient; t_Student_ = result of t-test for correlation

The results of optimal stratification analysis for finding cut-off values for SMI, VATI, SATI, VSR and MRA are presented in Table 3 and Supplementaly Figures 1–4.

### Survival analysis

#### Overall survival

In the cohort, median follow up time was 116 months, with 5-year OS of 66.8% (95% CI 53.9–82.7). The result of univariate analysis of OS is presented in Supplementaly Table 2. Of the nutritional and body composition features, myopenia (HR 3.18, 95% CI 1.11–8.56, p = 0.020), cancer cachexia (HR 6.07, 95% CI 2.24–16.46, p < 0.001), high VSR (HR 4.32, p = 0.043) and low SATI (HR 4.91, 95% CI 1.11–21.65, p = 0.02) were associated with elevated risk for overall mortality. SMI and BMI had small protective impact on OS in univariate analysis (HR 0.95, p = 0.040 and HR 0.83, p = 0.036, respectively). Of the known prognostic factors, preoperative levels of albumin, CRP, NLR, and AJCC stage were associated with OS. Major postoperative morbidity (CD > IIIa) was significantly correlated with shorter OS (HR 3.16, 95% CI, 1.24–8.04, p = 0.016). Multivariate analysis of OS confirmed the significance of myopenia (myopenic vs. non-myopenic: adjusted HR 6.5, p = 0.032), cancer cachexia (cachectic vs. non-cachectic: adjusted HR 13.7, p = 0.004) and high SATI (adjusted HR 7.00, p = 0.057). Major postoperative morbidity, NLR and albumin level also remained significant in multivariate OS analysis (Figure 2 A–E).

#### Local-recurrence free survival

The 5-year LRFS for whole study cohort was 77.6% (95% CI, 65.2–92). In univariate analysis among all body composition parameters, only SMI and SATI (low *vs*. high) showed association with LRFS (HR 0.94, 95% CI 0.88–1.00, p = 0.052 and HR 8.77, 95% CI 1.12–68.69, p = 0.039, respectively). Tumour size (HR 1.05, 95% CI 1.01–1.09, p = 0.016) and AJCC stage (3A–3B *vs*. 1A–1B, HR 4.46, 95% CI, 0.95–20.95, p = 0.058) were also associated with LRFS. However, SMI and SATI lost statistical significance in multivariate model, while tumour size and AJCC stage remained significant (Figure 2 F and Supplementaly Table 2).

### Postoperative outcome and morbidity

We performed univariate and multivariate risk factor analysis to evaluate factors associated with postoperative morbidity, intrahospital length of stay (LOS) and major postoperative complications.

The median postoperative LOS was 18 days (IQR: 11.25–28.75). In univariate analysis, higher ASA grade (2–3 *vs*. 1, OR 5.56, p = 0.025) and tumour size (OR 1.12, p = 0.007) showed correlation with prolonged LOS. Preoperative CRP (OR 1.02, p = 0.007), resection status (R1–R2 *vs*. R0 OR 4.87, p = 0.046) and intraoperative blood loss (OR 1.10, p = 0.065) were associated with major postoperative morbidity (Table 4 and Supplementaly Table 3).

**TABLE 4.** Summary of univariate and multivariate analysis of association between body composition and outcome following surgery for primary RPS

**Clinicopathological factor**	**Length of hospital stay (> 10 days)**				**Clavien-Dindo > IIIa**				**Any complication (overall morbidity)**			

	**Uni-variable**		**Multi-variable**		**Uni-variable**		**Multi-variable**		**Uni-variable**		**Multi-variable**	

	**OR (95% CI)**	**p**	**OR (95% CI)**	**p**	**OR (95% CI)**	**p**	**OR (95% CI)**	**p**	**OR (95% CI)**	**p**	**OR (95% CI)**	**p**
Myopenia, yes *vs*. no^a^	1.34 (0.38–5.54)	0.664	-	-	0.32 (0.07–1.18)	0.112	-	-	3.11 (0.85–15.08)	0.112	-	-
Visceral obesity, yes *vs*. no	2.54 (0.68–12.40)	0.196	-	-	0.65 (0.18–2.13)	0.49	-	-	3.61 (1.09–14.44)	0.047	-	-
Myopenic obesity, yes *vs*. no	0.01 (0.00–0.001)	0.993	-	-	20090605.83 (0.00–NA)	0.993	-	-	279.10 (0.00–NA)	0.993	-	-
Myosteatosis, yes *vs*. no	1.39 (0.39–5.76)	0.626	-	-	2.17 (0.58–10.58)	0.282	-	-	5.05 (1.39–24.41)	0.023	4.63 (1.03–28.42)	0.063
Cancer cachexia, yes *vs*. no	0.68 (0.18–2.94)	0.585	-	-	2.83 (0.76–10.59)	0.117	-	-	0.95 (0.27–3.60)	0.935	-	-
Body mass index, kg/m^2^	1.00 (0.89–1.14)	0.992	-	-	0.94 (0.81–1.06)	0.351	-	-	0.95 (0.27–3.60)	0.935	-	-
Skeletal Muscle Area, HU	1.00 (0.99–1.02)	0.869	-	-	1.00 (0.98–1.01)	0.679	-	-	1.00 (0.99–1.02)	0.681	-	-
Skeletal Muscle Index, cm^2^/m^2^	1.00 (0.95–1.07)	0.88	-	-	1.00 (0.95–1.06)	0.909	-	-	1.04 (0.98–1.10)	0.230	-	-
Muscle Radiation Attenuation, HU	0.98 (0.92–1.04)	0.526	-	-	0.99 (0.93–1.05)	0.775	-	-	0.94 (0.88–1.00)	0.076	-	-
SAT, cm^2^	1.00 (0.99–1.00)	0.336	-	-	1.00 (0.99–1.00)	0.48	-	-	1.00 (0.99–1.01)	0.866	-	-
VAT, cm^2^	1.00 (1.00–1.01)	0.769	-	-	1.00 (0.99–1.00)	0.622	-	-	1.00 (1.00–1.01)	0.205	-	-
VSR	1.55 (0.53–5.51)	0.456	-	-	1.09 (0.39–2.861)	0.861	-	-	1.72 (0.66–5.19)	0.292	-	-
High VSR^b^, yes *vs*. no	2.50 (0.63–9.52)	0.179	-	-	2.75 (0.63–19.26)	0.224	-	-	6.19 (1.69–26.52)	0.008	5.05 (1.08–29.74)	0.05
Body fat, %	0.96 (0.87–1.05)	0.380	-	-	1.00 (0.92–1.09)	0.962	-	-	1.00 (0.93–1.09)	0.912	-	-
Lean Body Mass, kg	1.01 (0.96–1.07)	0.765	-	-	1.02 (0.97–1.08)	0.381	-	-	1.02 (0.97–1.08)	0.381	-	-
SATI, cm^2^/m^2^	0.99 (0.97–1.01)	0.279	-	-	0.99 (0.97–1.01)	0.344	-	-	1.00 (0.99–1.02)	0.794	-	-
VATI, cm^2^/m^2^	1.00 (0.99–1.02)	0.625	-	-	1.00 (0.98–1.01)	0.706	-	-	1.01 (1.00–1.04)	0.122	-	-
High SATI^c^, yes *vs*. no	1.80 (0.52–6.25)	0.346	-	-	0.90 (0.26–2.93)	0.863	-	-	1.50 (0.49–4.81)	0.481	-	-
High VATI^d^, yes *vs*. no	0.49 (0.10–1.87)	0.327	-	-	0.38 (0.08–1.44)	0.184	-	-	2.83 (0.84–11.45)	0.111	-	-

OR = Odds Ratio; SAT = Subcutaneous Adipose Tissue Area; SATI = Subcutaneous Adipose Tissue Index; VAT = Visceral Adipose Tissue Area; VATI = Visceral Adipose Tissue Index; VSR = Visceral-to-subcutaneous adipose tissue area ratio;

a assessed by the European Working Group on Sarcopenia in Older People revised criteria from 2018;

b defined as VSR > 0.26;

c defined as SATI > 49.23 for males and SATI > 86.89 for females;

d defined as VATI > 61.38 for males and VATI > 25.55 for females

Only significant variables (p < 0.05) were included in multivariate analysis.

In univariate analysis of overall postoperative morbidity, the presence of myosteatosis (OR 5.05, p = 0.023), VO (OR 3.61, p = 0.047) and high VSR (OR 6.19, p = 0.008) were associated with significantly higher overall complication rate. Adjusted for other covariates in multivariate analysis, high VSR maintained significant impact (adjusted OR 5.05, p = 0.05). We omitted VO from multivariate analysis to avoid multicollinearity. Figure 3 (panels A–F) summarises our analysis of morbidity following surgery for primary RPS.

## Discussion

Our study provided new insight into the association between preoperative body composition and postoperative and oncologic outcome in primary RPS patients. We focused on evaluation of the significance of preoperative nutrition status-related syndromes. Furthermore, we examined the predictive value of SMI and MRA, measures of muscular quantity and quality, as well as, VATI, SATI and VSR, measures of adiposity, for possible clinical use in preoperative clinical assessment of patients diagnosed with this rare malignancy. To address the lack of literature and inconsistency in used body composition cut points, we used maximally selected rank statistics to defined cohort – specific cut point. This method incorporated follow-up time and time-to-event outcomes, dividing the patients into two groups with the most significant statistics between each other in term of survival.^56^

In our cohort, both myopenia and VO were associated with poorer OS. Patients with myopenia had 5-year OS of 33.7%, compared to significantly higher 78.3% 5-year OS for non-myopenic patients (p = 0.009). SMI predicted LRFS on univariate analysis and lost prognostic value in multivariate analysis.

These findings are in line with knowledge that sarcopenic surgical oncology patients are at greater risk for poor operative outcome because of underlying muscle mass loss which is an integral component of sarcopenia and also facilitates the impairment of muscle function and physical performance.^57^ Therefore we used the cut-off values for diagnosis of myopenia which are the component of diagnostic criteria and tools that define and characterize sarcopenia in EWGSOP2 Revised European Consensus.^48^ Our optimal fitting method analysis for establishing the cut-off value for defining low SMI (used for comparative and descriptive purposes) resulted in slightly different cut-off values: SMI < 49.21 cm^2^/m^2^ for males and females with BMI < 25, and SMI < 49.9 cm^2^/m^2^ for males with BMI ≥ 25 and SMI < 50.64 cm^2^/m^2^ for females with BMI ≥ 25. Both EWGSOP2 criteria for low SMI and our cut-off values were able to predict poor prognosis. It seems, that difference is generated because our cohort consisted of only patients with primary RPS with resectable disease rather than a heterogeneous cohort. We also found that SMI analysed as continuous variable was not able to predict poor outcome. This is another proof that in clinical practice SMI should be evaluated as body composition (myopenia) parameter defined with cut-off values below which the risk of poor prognosis is increased significantly, rather than discretional decrease.^58^

Several studies demonstrated the superior predictive value of myosteatosis to sarcopenia or myopenia for poor survival.^59,60,61^ Most of this data is founded on reports about patients operated on for gastrointestinal cancers. In our study cohort myosteatosis was also associated with greater overall complication rate (OR 5.05, 95% CI 1.39–24.41, p = 0.023) in univariate analysis, but it was not confirmed in multivariate analysis. Myosteatosis was not associated with OS, LRFS or postoperative outcome. However, recently a group of authors reported significant association between myosteatosis and major complication rate and OS in retroperitoneal and trunk soft tissue sarcoma.^29^ They used preoperative MRA as continuous variable to define myosteatosis, not providing any cut-off point for reference. We defined myosteatosis based on optimal cut point analysis for MRA, and determined cut-offs are comparable to most commonly used range of MRA cut-offs for myosteatosis.^62^

In order to evaluate obesity and the distribution of fat tissue, we calculated VAT, SAT and corresponding height-adjusted indexes VATI and SATI. We also considered BMI. Higher value of BMI (≥ 25) was not associated with oncologic or postoperative outcome. This is in line with number of studies suggesting that BMI is not reliable prognostic parameter for predicting perioperative outcome in cancer patients.^63,64,65^ Stratified for myopenia, comparison of the subgroups revealed that body fat and VATI were significantly higher in non-myopenic patients (median 31.1 *vs*. 28.1%, p = 0.024 and 42.3 *vs*. 20.6 cm^2^/m^2^, p = 0.025, respectively) (Table 2). Further on, we used VAT to assess VO applying the ESPEN/EASO criteria.^39^ VO predicted poorer OS and higher postoperative complication rate. VAT alone had no impact on OS or postoperative outcome. Recent study on soft tissue sarcoma patients reported identical findings.^29^ We considered VSR into adiposity analysis defining subgroup of patients with normal and high VSR (> 0.26) based on optimal cut-off analysis. In multivariate analysis VSR was an independent predictor for overall complication rate following surgery. High VSR group experienced significantly more complications compared to normal VSR group (33 (89.2%) *vs*. 4 (10.8%), p = 0.008). These results are comparable with previous reports in which VSR was superior to VAT as independent risk factor for death and local recurrence. ^34,35,58,66,67^ Linear regression analysis showed significant corelation between VSR and both VATI and fat mass (Figure 3 – panels G–H and Supplementaly Figure 5), confirming the importance of balance between visceral and subcutaneous adipose tissue. Recent studies demonstrated that predictive values of VSR for cardiovascular and metabolic disease incidence is superior to VAT.^34,36,37,68^ However, to our knowledge, only a few studies investigated the impact of VSR and VAT on survival and postoperative outcome in patients operated on for primary RPS.^29^ Our study underlined that high VSR is not only superior to VAT but also to BMI in predicting poor oncologic and peri-operative outcome. These findings suggest that VSR better estimates adipose tissue distribution and poses an additional difficulty for performing the surgery itself. High VSR is strong independent predictor for overall postoperative morbidity (multivariable-adjusted OR 5.05, p = 0.05). On the other hand, in the context of survival analysis, the multivariate regression model was not able to confirm the statistical significance of VSR (p = 0.068). This suggests that the impact of VSR on survival of RPS patients may be attenuated when considered alongside the broader set of predictors. One of the reasons may be the fact that, the presence of high VSR, as determined by specific gender-independent cut-off criteria, exhibited a statistically significant gender difference, with females having higher odds (OR = 4.5, p = 0.027) compared to males. Furthermore, we found a statistically significant difference in the distribution of SMI, between the two groups defined by VSR (high VSR *vs*. low VSR OR = 0.926, p = 0.047). Logistic regression model revealed significant association of SMI with a reduced odds of the specified outcome within the “high VSR” group. Based on our initial hypothesis that “high VSR” has a negative impact on survival, supported by univariate analysis, this implies that SMI (approximation of myopenia) may be a factor that mitigates the negative impact of “high VSR” on patient survival or serves as a positive influence, hence confounding the effect of VSR in multivariate settings. Further prospective studies need to be developed to confirm the importance of preoperative VSR for poor postoperative survival. In contrast to high VAT, low SATI, independently predicted poorer OS (adjusted HR 7.00, p = 0.057). Recent study reported similar protective effect of higher subcutaneous fat in RPS patients^29^, which confirms the known benefits of SATI in processes of carcinogenesis and metabolism.^30,69,70,71^

The multivariate analysis demonstrated that, when assessed as a continuous variable, albumin levels (HR 0.9, 95% CI 0.81–0.98, p = 0.019) and NLR (HR 1.4, 95% CI 1.06–1.75, p = 0.015) were independently associated with overall survival. This finding underscores the pivotal role of these inflammatory biomarkers in clinical practice and management of surgical oncology patients. Our results align with previous findings.^17,18,72,73,74,75,76,77^ Furthermore, our observation suggests that hypoalbuminemia identifies a high-risk cohort that may derive greater benefits from enhanced nutritional support preoperatively. The omission of descriptive statistical analysis for serum albumin and NLR, in term of patients' outcome, limits the depth of our data exploration.

Our study had some limitations. It was a single center report including relatively small number of patients which might influence the power of drown conclusions. Another weakness was the fact that we didn't assess all comorbidities in our analyses, as they were considered negligible in patients with soft tissue sarcoma. However, since our Institution is the only referral sarcoma center in Slovenia, having population of 2.1 million, our study cohort consisted of unique set of primary RPS patients eligible for curative surgery. We reported the most distinguishable, cohort – specific, cut points for CT measured body composition (muscle and adipose tissue) parameters in regard to long term prognosis. And finally, providing a unique and new insight into the association between preoperative body composition and postoperative and oncologic outcome in primary RPS patients was the main strength of the study.

## Conclusions

Patients with primary RPS are in a great risk for nutritional disorders for number of reasons such as: requirement for demanding abdominal surgery in their management, long preclinical history and tumour size.^4,10,18,78,79^ In our study cohort there was a high prevalence of myopenia (32.8%) and visceral obesity (36.2%). Myopenia, cancer cachexia and low SATI were strongly associated with poor OS. High VSR was strong independent predictor for overall postoperative morbidity. Additional prospective studies are required to substantiate the role of preoperative VSR as independent prognostic factor for postoperative survival. Our findings suggest that clinical nutrition interventions towards improving visceral adiposity and myopenia may benefit surgical and oncologic outcome in primary RPS patients.

## Supplementary Material

Supplementary Material
