# Supplementary material for: Influence of nutritional status and body composition on postoperative events and outcome in patients treated for primary localized retroperitoneal sarcoma
Source: Radiol Oncol. 2024 Feb 21;58(1):110–23. doi: 10.2478/raon-2024-0013 (PMC10878779; doi:10.2478/raon-2024-0013)
Supplement: Supplementary file 1 — Supplementary Material [file raon-2024-0013-sm.pdf]

Influence of nutritional status and body composition on postoperative events and outcome in patients treated for primary localized retroperitoneal sarcoma

Manuel Ramanovic, Marko Novak, Andraz Perhavec, Taja Jordan, Karteek Popuri, Nada Rotovnik Kozjek

Radiol Oncol 2024; 58(1): 110-123.

doi: 10.2478/raon-2024-0013

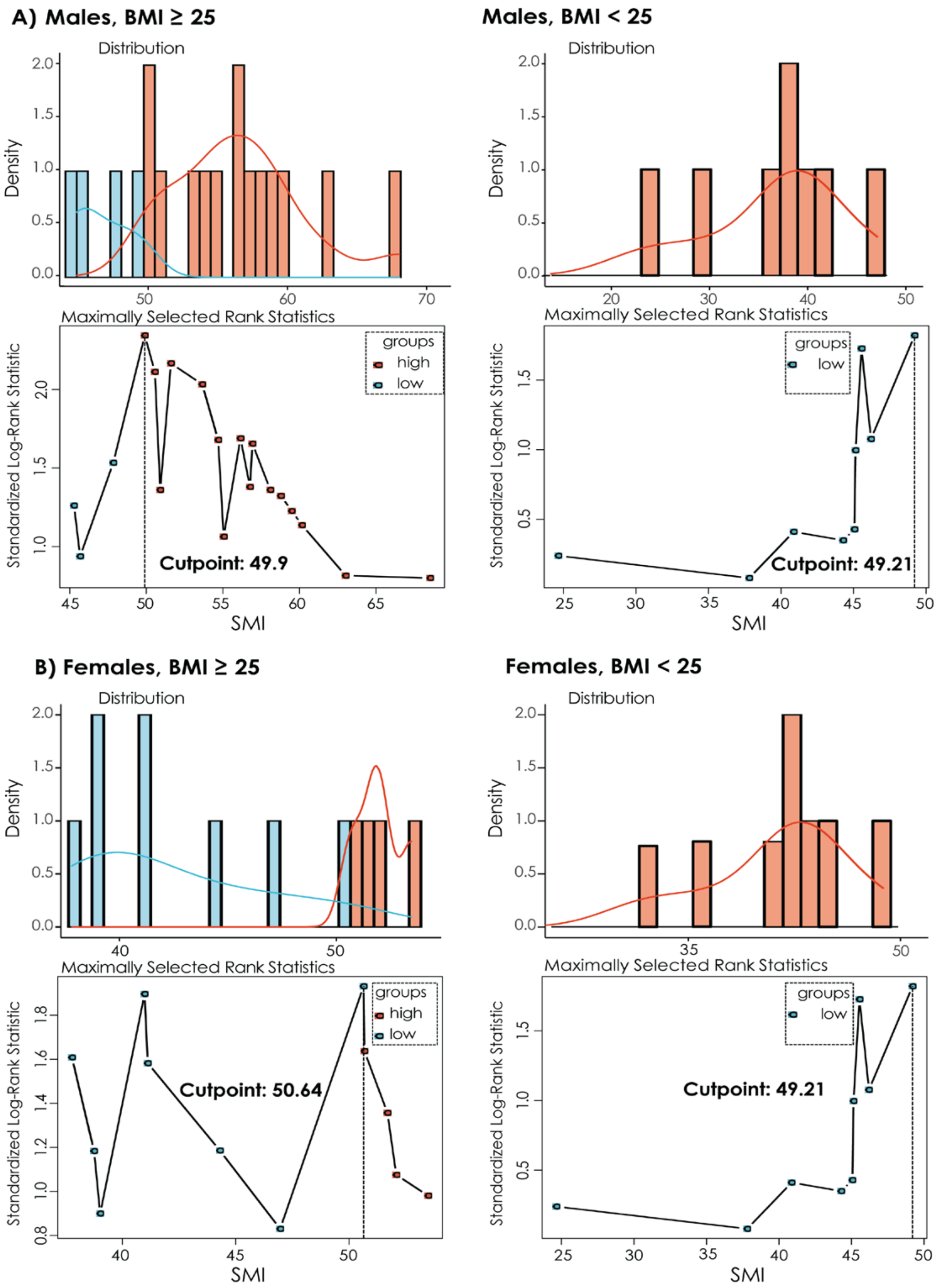

SUPPLEMENTALY FIGURE 1. Result of cut-off analysis for Skeletal muscle index adjusted to BMI for: **(A)** Males and **(B)** Females.

BMI = body mass index (kg/m^2); SMI = skeletal muscle index (cm^2/m^2).

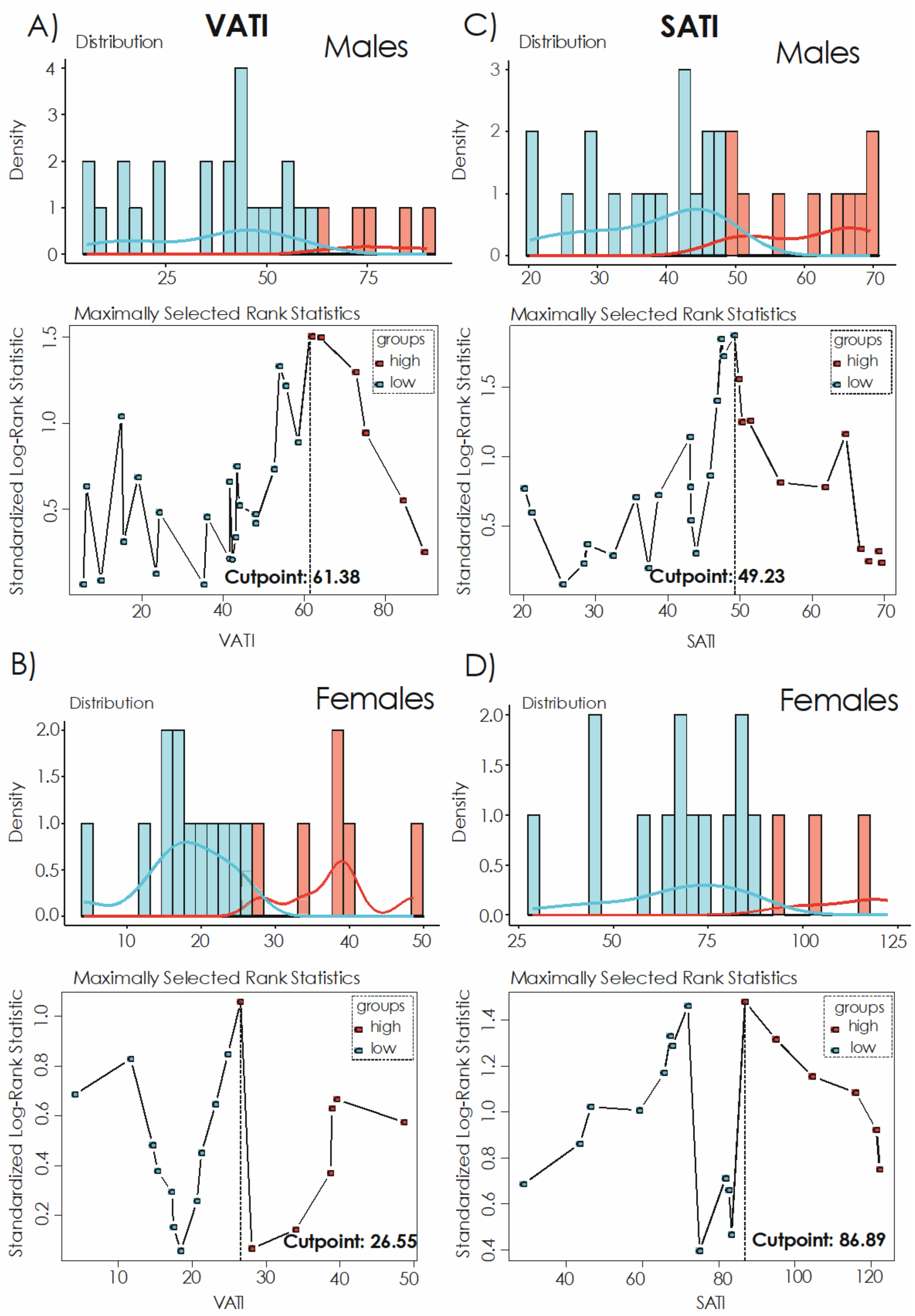

SUPPLEMENTALY FIGURE 2. Result of cut-off analysis for VATI (left) and SATI (right) for: males (**[A]** and **[C]**) and females (**[B]** and **[D]**).

SATI = subcutaneous adipose tissue index (cm^2/m^2); VATI = Visceral adipose tissue index (cm^2/m^2)

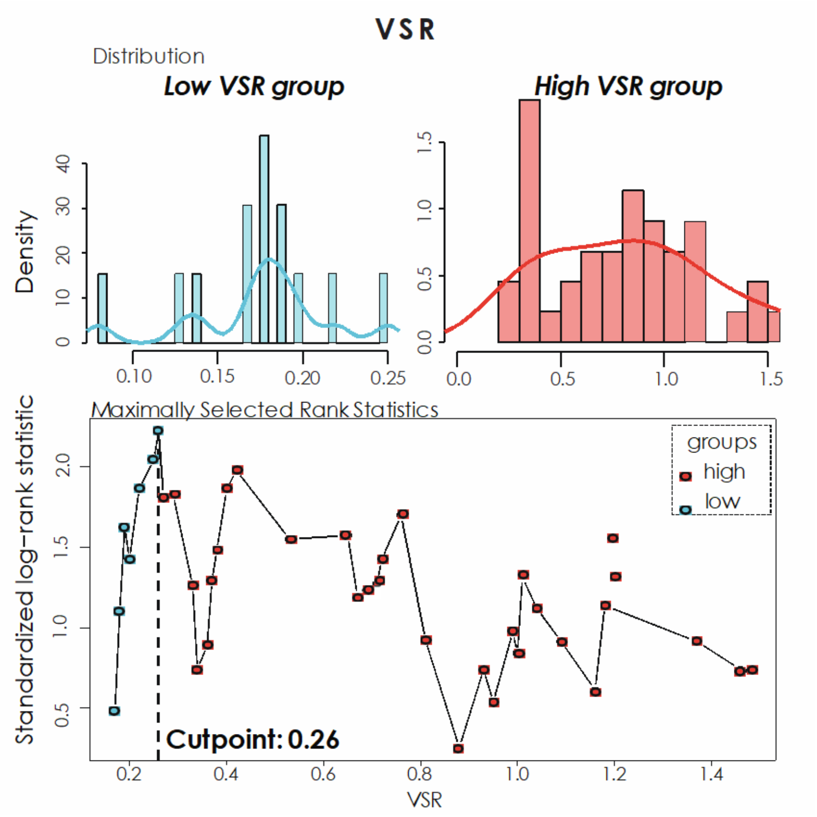

SUPPLEMENTALY FIGURE 3. Result of cut-off analysis for Visceral-to-subcutaneous adipose tissue area ratio: Cut point is 0.26, distribution of VSR values across low VSR and high VSR groups are presented.

VSR = Visceral-to-subcutaneous adipose tissue area ratio

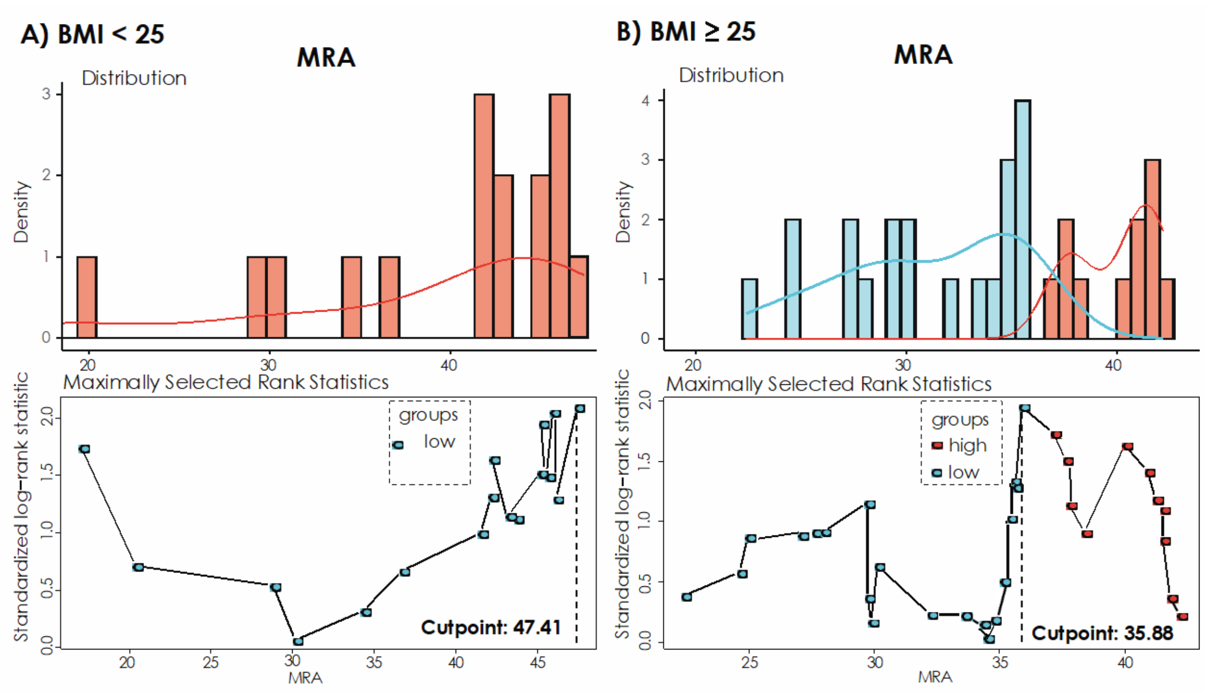

SUPPLEMENTALY FIGURE 4. Result of cut-off analysis for Muscle radiation attenuation, indicator of myosteatosis, adjusted to BMI: **(A)** BMI < 25; **(B)** BMI ≥ 25.

BMI = Body mass index (kg/m2); MRA= Muscle radiation attenuation (Hounsfield units)

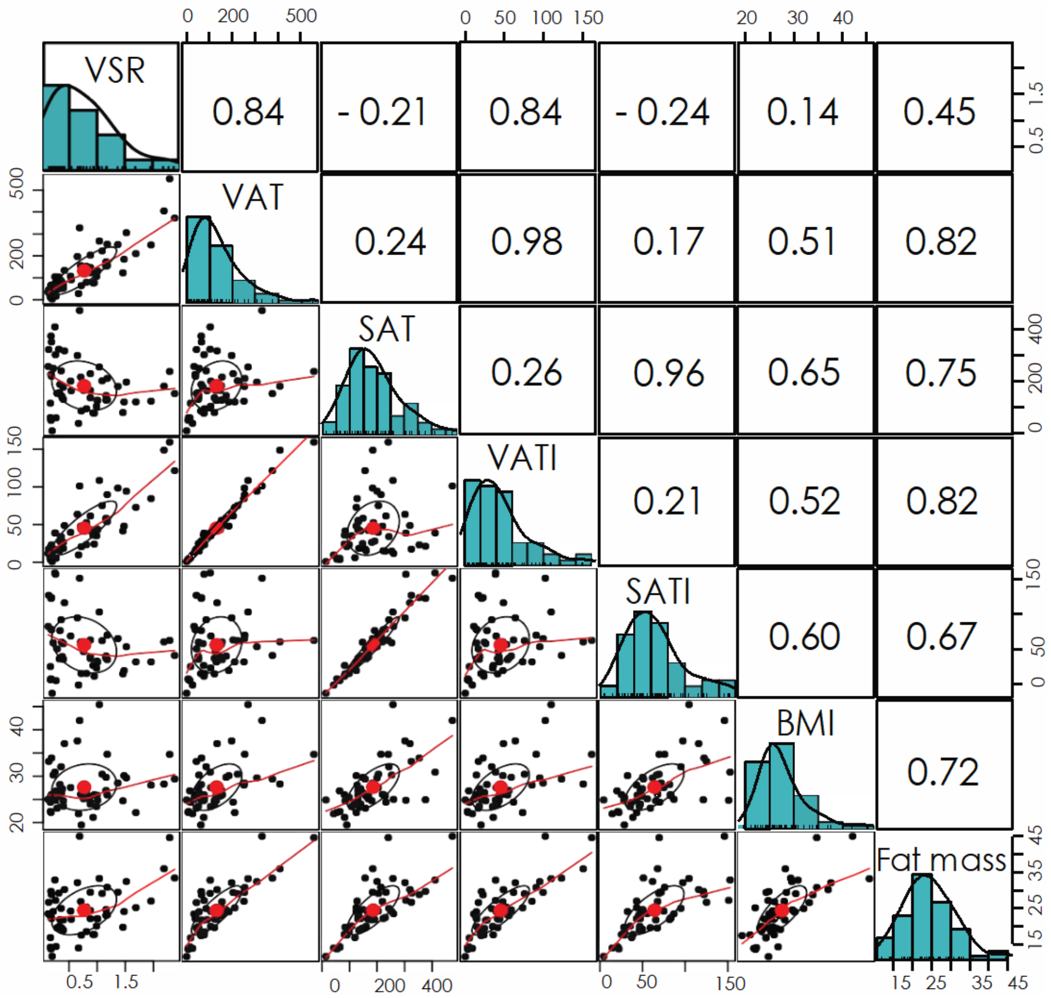

SUPPLEMENTALY FIGURE 5. Linear regression analysis of relationship between visceral-to-subcutaneous adipose tissue area ratio (VSR) and other adiposity related parameters. In upper off diagonal is reported Pearson correlation coefficient. Significant correlation is detected between VSR and visceral adipose tissue index and fat mass.

BMI = Body mass index (kg/m^2); SAT = Subcutaneous adipose tissue area (cm^2); SATI = Subcutaneous adipose tissue index (cm^2/m^2); VAT = Visceral adipose tissue area (cm^2); VATI = Visceral adipose tissue index (cm^2/m^2)

SUPPLEMENTALY TABLE 1. Comparison of clinical and body composition parameters between males and females

Clinicopathological factor	Level	Male	Female	p
Visceral obesity	No	22 (64.7)	15 (62.5)	1
	Yes	12 (35.3)	9 (37.5)	
Myopenic obesity	No	32 (94.1)	21 (87.5)	1
	Yes	2 (5.9)	2 (8.3)	
Myosteatosis	Yes	21 (61.8)	16 (66.7)	0.577
	No	13 (38.2)	6 (25.0)	
Cancer cachexia	No	21 (61.8)	22 (91.7)	0.003
	Yes	13 (38.2)	0 (0.0)	
Body Mass Index, kg/m^2	Mean (SD)	27.3 (4.5)	27.9 (5.8)	0.668
Skeletal Muscle Area, HU	Mean (SD)	163.7 (35.2)	120.0 (20.0)	< 0.001
Skeletal Muscle Index, cm^2/m^2	Mean (SD)	52.8 (10.6)	45.3 (8.2)	0.006
Muscle Radiation Attenuation, HU	Mean (SD)	36.8 (11.2)	37.7 (6.7)	0.731
Subcutaneous Adipose Tissue Area, cm^2	Mean (SD)	155.1 (84.6)	227.2 (92.6)	0.003
Visceral Adipose Tissue Area, cm2	Mean (SD)	153.9 (115.5)	96.0 (90.6)	0.045
Visceral-to-subcutaneous adipose tissue area ratio	Mean (SD)	1.0 (0.6)	0.5 (0.5)	0.001
Body fat, %	Mea (SD)	28.4 (6.1)	31.8 (8.0)	0.072
Lean Body Mass, kg	Mean (SD)	55.7 (7.1)	51.6 (17.5)	0.226
Subcutaneous Adipose Tissue Index, cm^2/m^2	Mean (SD)	49.8 (25.8)	85.0 (35.8)	< 0.001
Visceral Adipose Tissue Index, cm^2/m^2	Mean (SD)	49.7 (35.5)	36.5 (36.2)	0.183

SUPPLEMENTALY TABLE 2. Univariate and multivariate logistic regression analysis of factors associated with overall and local recurrence-free survival

			Overall survival		Local recurrence-free survival	
**Clinicopathological factor**	**Level**	**All**	**HR (univariable)**	**HR (multivariable)**	**HR (univariable)**	**HR (multivariable)**
Age, years	Mean (SD)	57.8 (15.0)	1.02 (0.98-1.05, p=0.278)		1.01 (0.97-1.05, p=0.530)	
Sex	Male	34 (58.6)	-		-	
	Female	24 (41.4)	0.43 (0.15-1.21, p=0.110)		0.57 (0.16-1.96, p=0.372)	
ASA Grade	1	9 (15.5)	-		-	
	2–3	49 (84.5)	4.37 (0.57-33.55,p=0.156)		2.50 (0.32-19.70,p=0.384)	
Tumour size, cm	Mean (SD)	21.5 (12.5)	1.02 (0.98-1.05, p=0.310)		1.05 (1.01-1.09, p=0.016)	1.05 (1.00-1.10, p=0.047)
Clavien-Dindo >IIIa	No	41 (70.7)	-	-	-	
	Yes	17 (29.3)	3.16 (1.24-8.04, p=0.016)	8.33 (1.58-43.78,p=0.012)	1.53 (0.45-5.23, p=0.501)	
Neutrophil-lymphocyte ratio	Mean (SD)	4.0 (3.0)	1.19 (1.05-1.36, p=0.009)	1.36 (1.06-1.75, p=0.015)	1.03 (0.79-1.34, p=0.845)	
Albumin, g/L	Mean (SD)	39.4 (8.2)	0.94 (0.88-0.99, p=0.022)	0.89 (0.81-0.98, p=0.019)	0.94 (0.87-1.01, p=0.085)	
C-reactive protein, mg/L	Mean (SD)	38.2 (47.0)	1.01 (1.00-1.02, p=0.003)	1.00 (0.99-1.02, p=0.742)	1.00 (0.99-1.01, p=0.812)	
Haemoglobin, g/L	Mean (SD)	126.2 (23.0)	0.98 (0.96-1.00, p=0.067)		1.00 (0.97-1.04, p=0.780)	
Preoperative radiotherapy	No	57 (98.3)	-		-	
	Yes	1 (1.7)	0.00 (0.00-Inf, p=0.998)		0.00 (0.00-Inf, p=0.998)	
Resection status	R0	47 (81.0)	-		-	
	R1	11 (19.0)	2.17 (0.80-5.85, p=0.127)		3.02 (0.87-10.49,p=0.082)	
Intraoperative blood loss, L	Mean (SD)	3.1 (5.6)	1.00 (1.00-1.00, p=0.002)	1.00 (1.00-1.00, p=0.977)	1.00 (1.00-1.00, p=0.157)	
Stage AJCC, 8th edition	1A–1B	24 (41.4)	-	-	-	
	3A–3B	34 (58.6)	4.22 (1.22-14.61,p=0.023)	1.16 (0.23-5.95, p=0.855)	4.46 (0.95-20.95,p=0.058)	5.13 (1.06-24.90,p=0.043)
Nutrition team before surgery	Yes	28 (48.3)	-			
	No	30 (51.7)	0.40 (0.14-1.15, p=0.090)			
Myopenia[a] (EWGSOP2 criteria)	No	19 (33.3)	-	-	-	
	Yes	38 (66.7)	3.18 (1.18-8.56, p=0.022)	6.54 (1.18-36.31,p=0.032)	2.69 (0.81-8.95, p=0.106)	
Visceral obesity	Yes	21 (36.2)	-		-	
	No	37 (63.8)	1.43 (0.46-4.42, p=0.540)		1.86 (0.39-8.78, p=0.432)	
Myopenic obesity	No	53 (93.0)	-		-	
	Yes	4 (7.0)	3.72 (0.45-30.75,p=0.223)		0.00 (0.00-Inf, p=0.999)	
Myosteatosis	Yes	37 (66.1)	-		-	
	No	19 (33.9)	0.78 (0.28-2.18, p=0.630)		0.76 (0.22-2.67, p=0.674)	
Cancer cachexia	No	42 (76.4)	-	-	-	
	Yes	13 (23.6)	6.07 (2.24-16.46,p<0.001)	13.72 (2.33-80.77,p=0.004)	2.64 (0.76-9.20, p=0.126)	
BMI	Mean (SD)	27.5 (5.0)	0.83 (0.71-0.99, p=0.036)		0.92 (0.78-1.07, p=0.283)	
Skeletal Muscle Area, HU	Mean (SD)	145.6 (36.7)	0.99 (0.97-1.00, p=0.130)		0.99 (0.97-1.01, p=0.433)	
Skeletal Muscle Index, cm^2/m^2	Mean (SD)	49.9 (10.3)	0.95 (0.90-1.00, p=0.040)		0.94 (0.88-1.00, p=0.052)	0.98 (0.90-1.06, p=0.549)
Muscle Radiation Attenuation, HU	Mean (SD)	37.2 (9.5)	1.01 (0.96-1.07, p=0.605)		1.02 (0.96-1.09, p=0.475)	

			Overall survival		Local recurrence-free survival	
**Clinicopathological factor**	**Level**	**All**	**HR (univariable)**	**HR (multivariable)**	**HR (univariable)**	**HR (multivariable)**
SAT, cm^2	Mean (SD)	184.9 (94.3)	1.00 (0.99-1.00, p=0.149)		0.99 (0.99-1.00, p=0.132)	
VAT, cm^2	Mean (SD)	129.9 (108.9)	1.00 (0.99-1.00, p=0.610)		1.00 (0.99-1.01, p=0.544)	
VSR	Mean (SD)	0.8(0.6)	1.14 (0.44-2.95, p=0.790)		1.03 (0.32-3.32, p=0.960)	
High VSR[b]	No	13 (22.4)	-		-	
	Yes	45 (77.6)	4.32 (0.94-19.80,p=0.043)	7.94 (0.86-73.14,p=0.068)	2.61 (0.54-12.57,p=0.233)	
Body fat, %	Mean (SD)	29.7 (7.0)	0.97 (0.90-1.04, p=0.375)		0.93 (0.85-1.02, p=0.144)	
Lean Body Mass, kg	Mean (SD)	54.1 (12.3)	1.00 (0.97-1.03, p=0.970)		1.03 (0.99-1.06, p=0.137)	
SATI, cm^2/m^2	Mean (SD)	63.7 (34.5)	0.98 (0.97-1.00, p=0.064)		0.98 (0.97-1.00, p=0.115)	
VATI, cm^2/m^2	Mean (SD)	44.5 (36.1)	0.99 (0.97-1.01, p=0.515)		0.99 (0.97-1.01, p=0.477)	
High SATI[c]	Yes	22 (39.3)	-	-	-	
	No	34 (60.7)	4.91 (1.11-21.65,p=0.02)	7.00 (0.95-51.78, p=0.057)	8.77 (1.12-68.69,p=0.039)	6.42 (0.65-63.92, p=0.113)
High VATI[d]	Yes	18 (32.1)	-		-	
	No	38 (67.9)	2.45 (0.55-10.95,p=0.241)		1.77 (0.37-8.34, p=0.472)	

ASA = American Society of Anesthesiologists; HR = Hazard ratio; SAT = subcutaneous adipose tissue area; SATI = subcutaneous adipose tissue index; VAT = visceral adipose tissue area; VSR = visceral-to-subcutaneous adipose tissue area ratio; VATI = visceral adipose tissue index;

[a] assessed by the European Working Group on Sarcopenia in Older People revised criteria from 2018; [b] defined as VSR > 0.26; [c] defined as SATI > 49.23 for males and SATI > 86.89 for females; [d] defined as VATI > 61.38 for males and SATI > 25.55 for females. Only significant variables ($p < 0.05$) were included in multivariate analysis.

SUPPLEMENTALY TABLE 3. Univariate and multivariate logistic regression analysis of factors associated with postoperative length of stay, major morbidity (Clavien-Dindo > IIIa) and overall postoperative morbidity

	Length of hospital stay (>10 days)				Clavien-Dindo > IIIa				Any complication (overall morbidity)			
	Univariable		Multivariable		Univariable		Multivariable		Univariable		Multivariable	
Clinicopathological factor	OR (95% CI)	p	OR (95% CI)	p	OR (95% CI)	p	OR (95% CI)	p	OR (95% CI)	p	OR (95% CI)	p
Age, > 65 vs. < 65 y/o	2.31 (0.61-11.30)	0.246			1.51 (0.46-4.86)	0.491			1.08 (0.35-3.47)	0.89		
Gender, female vs. male	0.92 (0.27-3.23)	0.897			0.70 (0.21-2.20)	0.545			1.24 (0.42-3.80)	0.702		
ASA Grade, 2–3 vs. 1	5.56 (1.24-26.76)	0.025	7.40 (1.20-63.26)	0.004	3.88 (0.63-75.15)	0.219			8.75 (1.85-63.82)	p=0.012	3.63 (0.61-30.79)	0.182
Tumour size, cm	1.12 (1.04-1.22)	0.007	1.12 (1.04-1.22)	0.006	1.02 (0.97 -1.06)	0.488			1.03 (0.98-1.08)	0.279		
Clavien-Dindo, > IIIa vs. ≤ IIIa	3.10 (0.72-21.63)	0.171			N.A.	N.A.			N.A.	N.A.		
Neutrophil-lymphocyte ratio	1.05 (0.86-1.40)	0.686			1.09 (0.90-1.34)	0.372			1.09 (0.90-1.41)	0.442		
Baseline albumin, g/L	0.97 (0.89-1.05)	0.48			0.96 (0.89-1.03)	0.256			0.97 (0.91-1.04)	0.458		
Baseline C-reactive protein, mg/L	1.01 (0.99-1.03)	0.258			1.02 (1.01-1.03)	0.007	1.01 (1.00-1.03)	0.064	1.00 (0.99-1.02)	0.603		
Haemoglobin level, g/L	0.99 (0.96-1.02)	0.602			0.99 (0.96-1.01)	0.314			0.99 (0.96-1.01)	0.314		
Resection status, R1–2 vs. R0	0.81 (0.20-4.20)	0.787			3.93 (1.00-16.18)	0.05	4.87 (1.04-24.57)	0.046	7.41 (1.26-141.58)	0.066		
Intraoperative blood loss, ml	1.00 (1.00-1.00)	0.136			1.10 (1.00-1.20)	0.028	1.10 (1.00-1.20)	0.065	1.00 (1.00-1.00)	0.046	1.00 (1.00-1.00)	0.284
Stage AJCC, 3A-3B vs. 1A-1B	1.59 (0.47-5.44)	0.454			3.10 (0.92-12.46)	0.083			2.03 (0.69-6.17)	0.203		
Nutrition team, no vs. yes	0.75 (0.21-2.51)	0.642			1.07 (0.34-3.38)	0.905			1.29 (0.44-3.84)	0.638		
Myopenia, yes vs. no[a]	1.34 (0.38-5.54)	0.664			0.32 (0.07-1.18)	0.112			3.11 (0.85-15.08)	0.112		
Visceral obesity, yes vs. no	2.54 (0.68-12.40)	0.196			0.65 (0.18-2.13)	0.49			3.61 (1.09-14.44)	0.047		
Myopenic obesity, yes vs. no	0.01 (0.00-0.001)	0.993			20090605.83 (0.00-NA)	0.993			279.10 (0.00-NA)	0.993		
Myosteatosis, yes vs. no	1.39 (0.39-5.76)	0.626			2.17 (0.58-10.58)	0.282			5.05 (1.39-24.41)	0.023	4.63 (1.03-28.42)	0.063
Cancer cachexia, yes vs. no	0.68 (0.18-2.94)	0.585			2.83 (0.76-10.59)	0.117			0.95 (0.27-3.60)	0.935		
Body mass index, kg/m^2	1.00 (0.89-1.14)	0.992			0.94 (0.81-1.06)	0.351			0.95 (0.27-3.60)	0.935		
Skeletal Muscle Area, HU	1.00 (0.99-1.02)	0.869			1.00 (0.98-1.01)	0.679			1.00 (0.99-1.02)	0.681		
Skeletal Muscle Index, cm^2/m^2	1.00 (0.95-1.07)	0.88			1.00 (0.95-1.06)	0.909			1.04 (0.98-1.10)	0.23		
Muscle Radiation Attenuation, HU	0.98 (0.92-1.04)	0.526			0.99 (0.93-1.05)	0.775			0.94 (0.88-1.00)	0.076		
SAT, cm^2	1.00 (0.99-1.00)	0.336			1.00 (0.99-1.00)	0.48			1.00 (0.99-1.01)	0.866		
VAT, cm^2	1.00 (1.00-1.01)	0.769			1.00 (0.99-1.00)	0.622			1.00 (1.00-1.01)	0.205		
VSR	1.55 (0.53-5.51)	0.456			1.09 (0.39-2.861)	0.861			1.72 (0.66-5.19)	0.292		
High VSR[b], yes vs. no	2.50 (0.63-9.52)	0.179			2.75 (0.63-19.26)	0.224			6.19 (1.69-26.52)	0.008	5.05 (1.08-29.74)	0.05
Body fat, %	0.96 (0.87-1.05)	0.38			1.00 (0.92-1.09)	0.962			1.00 (0.93-1.09)	0.912		

	Length of hospital stay (>10 days)				Clavien-Dindo > IIIa				Any complication (overall morbidity)			
	Univariable		Multivariable		Univariable		Multivariable		Univariable		Multivariable	
**Clinicopathological factor**	**OR (95% CI)**	**p**	**OR (95% CI)**	**p**	**OR (95% CI)**	**p**	**OR (95% CI)**	**p**	**OR (95% CI)**	**p**	**OR (95% CI)**	**p**
Lean Body Mass, kg	1.01 (0.96-1.07)	0.765			1.02 (0.97-1.08)	0.381			1.02 (0.97-1.08)	0.381		
SATI, cm^2/m^2	0.99 (0.97-1.01)	0.279			0.99 (0.97-1.01)	0.344			1.00 (0.99-1.02)	0.794		
VATI, cm^2/m^2	1.00 (0.99-1.02)	0.625			1.00 (0.98-1.01)	0.706			1.01 (1.00-1.04)	0.122		
High SATI[c], yes vs. no[c]	1.80 (0.52-6.25)	0.346			0.90 (0.26-2.93)	0.863			1.50 (0.49-4.81)	0.481		
High VATI[d], yes vs. no	0.49 (0.10-1.87)	0.327			0.38 (0.08-1.44)	0.184			2.83 (0.84-11.45)	0.111		

ASA = American Society of Anesthesiologists; OR = odds ratio; SAT = subcutaneous adipose tissue area; VAT = visceral adipose tissue area; VSR = visceral-to-subcutaneous adipose tissue area ratio; SATI = subcutaneous adipose tissue index, VATI = visceral adipose tissue index;

[a] assessed by the European Working Group on Sarcopenia in Older People revised criteria from 2018; [b] defined as VSR > 0.26;
[c] defined as SATI > 49.23 for males and SATI > 86.89 for females; [d] defined as VATI > 61.38 for males and SATI > 25.55 for females.
Only significant variables ($p < 0.05$) were included in multivariate analysis.
